# Dietary propolis improves the growth performance, redox status, and immune response of Nile tilapia upon a cold-stress challenge

**DOI:** 10.1371/journal.pone.0293727

**Published:** 2023-11-02

**Authors:** Yousof N. Alrashada, Hesham A. Hassanien, Ahmed O. Abbas, Sami A. Alkhamis, Akram I. Alkobaby

**Affiliations:** 1 Department of Animal and Fish Production, College of Agriculture and Food Sciences, King Faisal University, Hofuf, AL-HASA, Saudi Arabia; 2 Animal Production Department, Faculty of Agriculture, Cairo University, Giza, Egypt; Tamil Nadu Dr J Jayalalithaa Fisheries University, INDIA

## Abstract

The purpose of this research was to demonstrate the potential of adding propolis (PR) to the diet of Nile tilapia (*Oreochromis niloticus*) to mitigate the harmful effect of cold stress (CS) on the growth performance, redox status, and immunological response. Two trials were conducted in this study. First, 210 Nile tilapia fingerlings (28.61±0.20 g) were used in a preliminary trial to determine the appropriate PR level and supplementation period to be applied for the main trial. Fish were assigned into 7 treatment groups (3 aquaria replicates × 10 fish per aquarium in each treatment group) according to the rate of PR supplementation in the fish diets at 0, 2, 4, 6, 8, 10, and 12 g/kg for 6 consecutive weeks. The average body weight and body weight gain were determined weekly. It was found that PR supplementation at 10 g/kg in fish diet for 4 weeks was enough to obtain significant results on the growth performance of Nile tilapia. For the main trial of the present study, 480 Nile tilapia fingerlings (average weight 29.93±0.11 g) were distributed into randomized 2 PR × 2 CS factorial treatment groups (6 replicate aquariums containing 20 fish in each group). Fish of PR groups received a basal diet for a feeding period of 4 weeks, included with 10 g/kg PR (+ PR group) or without PR inclusion (- PR group). Fish of the CS groups were either challenged with cold stress at 18°C (+ CS group) or maintained at a temperature of 26°C during the feeding period (- CS group). The results showed that CS challenge significantly (p < 0.05) impaired the growth indices, redox status, and immune response in the challenged fish compared to the non-challenged fish. On contradictory, the inclusion of PR into fish diets enhanced (p < 0.05) the feed intake, growth indices, antioxidant enzyme activity, and immunological parameters. Moreover, PR treatment alleviated the CS deterioration of fish weights, specific growth rates, feed efficiency, antioxidant enzyme activity, lymphocyte proliferation, and phagocytosis activity and alleviated the elevated mortality, H/L ratio, and malondialdehyde levels by cold stress. It is concluded that the inclusion of propolis at 10 g/kg in the diet of Nile tilapia fish could be approved as a nutritional approach to enhance their performance, especially when stressed by low-temperature conditions.

## Introduction

Nile tilapia (*Oreochromis niloticus*) has been a vital species in the effort to fulfill the rising demand for aquatic products in the world population [1]. According to the recent FAO report [2], the global tilapia market is expected to reach US$ 9.2 billion by 2027 and achieve an annual growth rate of 2.2% in the coming few years. About 26.9% of the tilapia global production is farmed in China, followed by other countries such as Indonesia (20.3%), Egypt (17.4%), Brazil (5.3%), the Philippines (4.6%), and Thailand (3.5%) [3]. It is a popular warm-water aquaculture fish due to its rapid development, high feed utilization, natural reproduction, and tasty flesh [4]. However, Nile tilapia fish have a thermal preference of 25–30°C and are very susceptible to cold stress [5]. Several studies have reported that winter cold fronts frequently cause a considerable economic drop in tilapia production and survival rates [6, 7]. It was also reported that temperatures below 20°C induce a noticeable physiological disturbance, metabolic dysfunctions, and immunological abnormalities, leading to a significant decline in growth performance and feed efficiency [8–10].

Over the years, significant advances in fish nutrition have led to commercial, well-balanced diets that promote optimal growth and the harvest of impeccable fish [11]. The use of medicinal and fragrant plants in nutrition has been considered one of the novel strategies for increasing fish resistance to adverse environmental conditions and various stressors [12–14]. In addition, including products from beehives in fish diets has gained great attention because of their low environmental impact and low cost [15]. Among these bee products is propolis (PR), which contains essential oils, waxes, and plant resinous gathered by honeybees [16]. PR comprises over 200 bioactive materials, including phenolic and flavonoid compounds [17–19]. PR is known to have other beneficial characteristics, such as antioxidant properties [20], immunomodulation [21], antiinflammation [22], and antimicrobial activity [23].

Previous research has investigated the effects of propolis supplementation at different levels and forms on fish diets. For example, Abd-El-Rhman [24] found that feeding Nile tilapia with 1% (10 g/kg) propolis-ethanolic extract or crude propolis enhanced growth, immunity, and bacterial resistance. Also, Abdel Mageid et al. [25] reported that propolis at 2.5 g/Kg or nano-propolis at 1.25 g/kg has an ameliorative effect against oxidative stress induced by *Microcystis aeruginosa* in the Nile tilapia fish. Moreover, Hassaan et al. [6] found linear mitigation of winter thermal stress by increasing dietary propolis-extract levels (1–4 g/kg) in Nile tilapia. Furthermore, it was found that propolis can moderate the growth performance and immunological response in other fish species [15, 26–29].

According to our knowledge, the beneficial effects of propolis on aquaculture have not been fully recognized and need to be investigated, especially under cold stress conditions. Therefore, two trials were carried out in the present research. The first trial was a preliminary study aimed to determine the appropriate propolis (PR) level and supplementation period that induce notable effects on the growth performance of Nile tilapia. Then, the second trial was conducted to evaluate if the selected dietary treatment with PR can alleviate the deterioration effect of cold stress on the growth aspects, redox status, and immunological response in Nile tilapia.

## Materials and methods

### Ethical statement

The current study protocol was authorized by the ethical research committee of Saudi Arabia’s King Faisal University (Approval no. KFU-REC-2023-APRIL-ETHICS95).

### Propolis analysis

The PR was gathered as a yellow-brown powder from a beehive station at the King Faisal University of Saudi Arabia’s Agricultural and Veterinary Research Center. The chemical characteristics of three PR samples were examined using the AOAC-adopted techniques [30]. The PR total polyphenolic and flavonoid concentrations were also assayed using the Folin-Ciocalteu and aluminum chloride colorimetric techniques. Gallic acid and quercetin were used as standards for each assay [31]. According to prior research [32], the 2,2-diphenyl-1-picrylhydrazyl (DPPH) radical scavenging assay was used to identify the PR’s antioxidant activity.

### Experimental design

**Fig 1:**
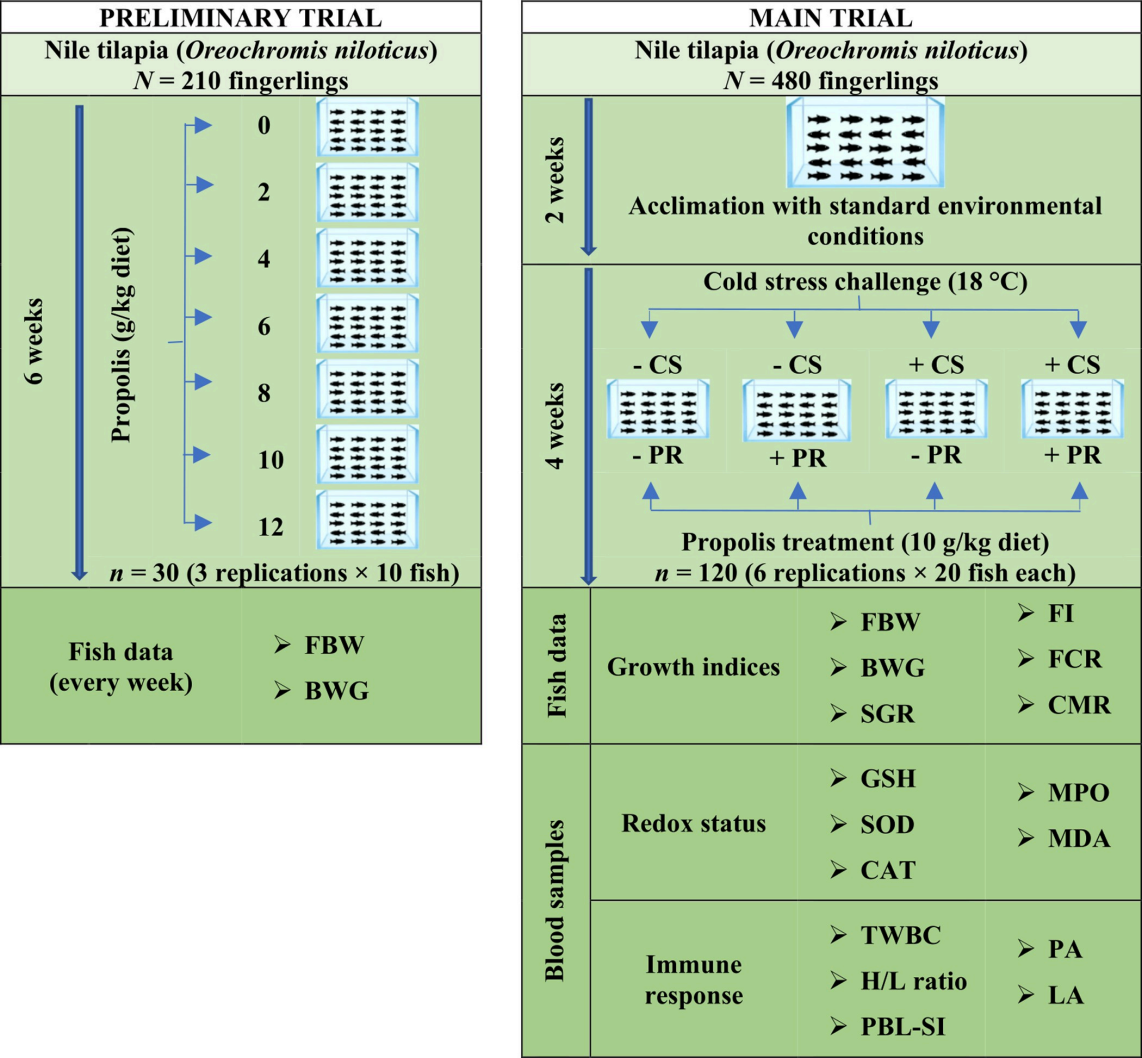
Schematic diagram explaining the experimental design of the present study. Abbreviations:—CS, fish group that was not challenged by cold stress; + CS, fish group that was challenged by cold stress;—PR, fish group that received a basal diet with no propolis supplementation; + PR, fish group that received a basal diet with 10 g/kg propolis; FBW, final body weight; BWG, body weight gain; SGR, specific growth rate; FI, feed intake; FCR, feed conversion ratio; CMR, cumulative mortality rate; GSH, reduced glutathione; SOD, superoxide dismutase; CAT, catalase; MPO, myeloperoxidase; MDA, malondialdehyde; TWBC, total white blood cells; H/L ratio, heterophil to lymphocyte cell ratio; PBL-SI, peripheral blood leukocyte stimulation index; PA, phagocytic activity; LA, lysozyme activity.

Two trials were carried out in the present study, as shown in Fig 1. First, a preliminary experiment was carried out on 210 Nile tilapia (*Oreochromis niloticus*) to determine the rate of PR that induce notable effects on the growth performance of Nile tilapia. Fish fingerlings (28.61±0.20 g) were obtained from a private aquaculture corporation (Saqua Co., Riyadh, Saudi Arabia) and reared into 21 aquaria (10 fish per aquarium) under standard environmental conditions for Nile tilapia (26°C, 6.5 mg/L dissolved O_2_, and 7.5 pH). The aquaria were divided into 3 replicates × 7 treatment groups according to the rate of PR supplementation in the fish diets at 0, 2, 4, 6, 8, 10, and 12 g/kg. Meals were introduced twice a day and calculated weekly on 3% of the fish biomass for 6 consecutive weeks. The average body weight and body weight gain were determined weekly and statistically analyzed to select the appropriate PR level and supplementation period for conducting the main trial in the present study. For the main trial, four hundred eighty Nile tilapia fingerlings with average weight of 29.93±0.11 g were obtained from the same private aquaculture corporation (Saqua Co., Riyadh, Saudi Arabia). First, fish fingerlings were acclimated for 2 weeks before starting the trial by keeping them in a 1 m^3^ capacity glass aquarium provided with standard environmental conditions for fish aquaculture [33]. After that, fish were distributed into totally randomized 2 PR × 2 CS factorial treatment groups (6 replicate aquariums per group; each aquarium volumed 120 L and contained 20 fish). The fish in the PR groups were either given a basal diet with no PR supplementation (- PR group) or a basal diet with 10 g/kg PR (+ PR group). Feeding of diets in each group was done twice a day (at 8:00 a.m. and 3:00 p.m.) for 4 weeks. The quantity of feed introduced was adjusted every week at a rate of 3% of the total fish biomass in each aquarium. Fish of CS groups were either challenged with a cold stress of 18°C (+ CS group) or maintained at a temperature of 26°C during the feeding period (- CS group). The cold stress was induced by a thermostat attached to a cooling system that gradually reduced the water temperature by 1°C/12h until 18°C [12].

About 25% of the aquarium water was siphoned while collecting feed remains every day and immediately compensated with aerated freshwater pre-adjusted at the same temperatures. Besides, the aquaria were cleaned from the wastes, and 50% of the aquarium water was exchanged with aerated freshwater at 3-day intervals during the experimental period. The diet components were properly combined and compacted into 1.5 mm diameter pellets using a pellet mill equipment. The diet pellets were then dried at room temperature for 24 hours before being kept in a refrigerator at 4°C. Table 1 shows the basal diet’s contents and the nutritional analysis performed by AOAC techniques [30].

**Table 1:** The composition and chemical analysis of the basal diet introduced to Nile tilapia fish.

Components	g/kg as fed	Nutritional analysis	g/kg (DM basis)
**Soybean meal**	320.0	**Dry matter (DM)**	908.0 ± 4.62
**Yellow corn**	315.0	**Crude protein (CP)**	349.8 ± 1.51
**Fish meal**	180.0	**Crude lipids (CL)**	68.3 ± 4.62
**Wheat bran**	60.0	**Crude fiber (CF)**	30.7 ± 0.32
**Corn gluten**	52.0	**Total ash (TA)**	75.0 ± 0.58
**Vegetable oil**	50.0	**Nitrogen-free extract (NFE)** ^**2**^	476.2 ± 7.91
**Premix** ^**1**^	14.0	**Gross energy (GE)** ^**3**^	19.2 ± 0.23
**Di-calcium phosphate**	5.0		
**Methionine**	2.0		
**Lysin**	2.0		

^1^ Contents per kg of premix: 40000 IU retinol, 4000 IU cholecalciferol, 400 mg α-tocopherol acetate, 12 mg menadione, 30 mg thiamine, 40 mg riboflavin, 30 mg pyridoxine, 80 μg cyanocobalamin, nicotinic acid, 10 mg folic acid, 3 mg biotin, 100 mg pantothenic acid, 500 mg inositol, and 500 mg ascorbic acid, 40 mg manganese sulfate, 10 mg magnesium oxide, 40 mg potassium sulfate, 60 mg zinc carbonate, 0.4 mg potassium iodide, 12 mg Copper sulfate, 250 mg ferric citrate, 0.24 mg sodium selenite, 0.2 mg cobalt.

^2^ NFE = 1000 –(CP + CL + CF + TA).

^3^ GE (MJ/kg) = (23.63 CP + 39.52 CL + 17.15 NFE) / 1000.

### Growth indices

For calculating feed intake (FI), the uneaten feed was collected, dried, and deducted from the introduced quantity, which was adjusted as mentioned before. Fish body weights per replication were noted at the start and end of the feeding period (4 weeks) to determine the initial (IBW) and final (FBW) body weights. The growth indices were calculated per replicate in each treatment group as in the following formulas: BWG = FBW–IBW, where BWG = body weight gain, FBW = final body weight, and IBW = initial body weight; SGR = 100×((Ln (FBW)–Ln (IBW))/D), where SGR = specific growth rate, Ln = natural logarithm, FBW = final body weight, IBW = initial body weight, and D = days of the feeding period; and FCR = FI/BWG, where FCR = feed conversion ratio, FI = feed intake, and BWG = body weight gain. The number of dead fish per replicate was recorded daily during the feed period for each group to evaluate the cumulative mortality rate (CMR) according to the following equation (CMR = the total number of dead fish/ the initial number of fish × 100).

### Redox status

Blood samples were taken from the caudal vein of 3 fish per replication, in each treatment group, after the feeding period (4 weeks), after the fish had been given anesthesia by submersion in water containing 100 mg/L tricaine methane sulfonate (MS-222) [34]. Blood samples were quit overnight at 4°C to coagulate. The serum was harvested by a 20 min centrifugation at 1075 × g and then kept at −20°C until later analysis of redox status. The reduced glutathione (GSH) in the sample was determined indirectly through its reaction with dinitrobenzoic acid (DNBT) and releasing a yellow complex which can be measured by colorimetric assay at 405 nm. The serum’s superoxide dismutase (SOD) was assayed based on the SOD inhibitory effect on the nitrite formation from hydroxylamine oxidation by the xanthine-xanthine oxidase reaction system. The nitrite could be turned purple by adding a chromogenic reagent and finally measured calorimetrically at 550 nm [35]. The catalase (CAT) activity was also assayed based on the principle that CAT decomposes H_2_O_2_. Then the residual H_2_O_2_ reacts with ammonium molybdate forming a yellowish complex that can be measured at 405 nm [36]. In contrast, myeloperoxidase (MPO) was measured based on reducing hydrogen peroxide to a complex that reacts with o-dianisidine and produces a yellowish color read at 460 nm [37]. In addition, the malondialdehyde (MDA) was assayed through its reaction with thiobarbituric acid (TBA) and then produced a red compound that can be measured calorimetrically at 532 nm [38]. All assays were performed according to the manufacturer’s instructions of colorimetric kits available from Elabscience Biotechnology Inc. (Houston, Texas, USA) and using an automated microplate reader (ELx808TM BioTek Instruments, Winooski, Vermont, USA).

### Immunological response

#### TWBC and H/L determination

Three blood samples per replicate in each treatment group (18 samples in total) were obtained after the feeding period (4 weeks) and gently shaken into tubes containing an anticoagulant (10% ethylenediaminetetraacetate; EDTA). A drop of the complete blood sample was allocated to count the total white blood cells (TWBC) by using a Bright-Line™ hemocytometer (American Optical, Buffalo, NY, USA), according to conventional techniques previously published by Rawling et al. [39]. In addition, another drop of blood was allocated to discriminate a total of 200 counted leukocyte cells, including the heterophils (H) and lymphocytes (L), by using Hema-3 stain solutions (Fisher Scientific, Pittsburg, PA, USA). Consequently, the H/L ratio was determined [40].

#### PBL proliferation assay

The remaining portion of the previous liquified blood samples was used to carry out the peripheral blood leukocyte (PBL) proliferation assay, according to the methods described by Carvalho et al. [41] with a minor modification. Briefly, blood samples were diluted 1:2 with PBS, stacked on a density gradient medium (Histopaque-1077, Sigma, MA, USA), and then centrifuged at 400 × g for 30 min at room temperature. The cell layer formed on the interface was carefully aspirated and washed twice with sterile PBS by 10 min centrifugation at 600 × g. The cell pellet from this process was resuspended in 1 mL of RPMI-1640 complete culture medium (Invitrogen Corp., Grand Island, NY, USA), enumerated and checked for > 95% viability using trypan blue dye. Viable lymphocytes were re-adjusted to a concentration of 3×106 cells/mL and distributed into 3 volumes in 96-well U-bottom microplates. Lipopolysaccharide solution at 10 μg/mL (LPS, Sigma, MA, USA) was added to the cell suspension to promote PBL stimulation. A set of wells were supplemented with RPMI-1640 to serve as control cells. The microplate wells were incubated for 18 h at 27°C and then incubated with tetrazolium salt MTT (3-(4,5-dimethyl-2thiazolyl)-2,5-diphenyl tetrazolium bromide) for a further 4 h. The plate was centrifuged at 110 × g for 5 min, the supernatant was removed, and the precipitated formazan was dissolved again with 100 mL dimethyl sulfoxide (DMSO). The optical density (OD) at 570 nm for the experimental and control wells was recorded using an automated microplate reader (Bio-Rad 550 Laboratories Inc., USA). Finally, the PBL proliferation capacity was calculated and represented as stimulation index (SI = OD stimulated cells ÷ OD non-stimulated control cells).

#### Phagocytosis assay

The phagocytic activity (PA) was assayed following the procedures of Almarri et al. [42], with some modifications. Briefly, blood samples were collected from fish in each group (18 samples) after the feeding period (4 weeks), and the leukocyte suspension was isolated using a separation medium, as previously mentioned. The recovered leukocyte suspension was mixed with a yeast suspension (*Candida albicans*, Sigma, MA, USA), which was previously stained with Tetramethyl-Rhodamine-Isothiocyanate (TRITC) dye at a ratio of 1:4 and incubated in a 24-well gelatin-plasma coated plates at 37°C for 30 min. The total phagocytes and those with at least one yeast were enumerated using a hemocytometer and inverted microscope. The PA (%) of leucocytes = [(number of phagocytes with engulfed yeast/ total phagocytes) × 100].

#### Lysozyme assay

This test relies on the potential of lysozyme proteins existing in the fish blood to break down the cell wall of Gram-positive bacteria. The lysozyme activity (LA) was determined using the turbidimetric technique described in a previous study [29]. In brief, 1 mL of *Micrococcus lysodeikticus* suspension (Sigma-Aldrich, Burlington, MA, USA) was mixed with 50 μL of Nile tilapia fish serum (18 samples per group). Using a spectrophotometer (CE1010, Cecil Instruments Limited, Cambridge, United Kingdom), the reduction in absorbance at 0.5- and 4.5-min intervals for 30 min was observed at a wavelength of 450 nm. The drop in absorbance by 0.001 per minute was used to define one unit of LA.

### Statistical analysis

The statistical analysis software, IBM SPSS Statistics version 22 (IBM Corp., NY, USA), was used to explore the results of the preliminary experiment and the main trial. In the preliminary experiment, the data of body weights and body weight gains for fish that received various levels of propolis (0, 2, 4, 6, 8, 10, and 12 g/kg diet) for 6 consecutive weeks were analyzed using one-way analysis of variance (ANOVA) with a polynomial contrast test to explore the linear and quadratic trends of the increased PR levels. After that, a 2 × 2 factorial design was used to organize the data of the main trial in the present study, considering the main components in this study as PR treatment of 10 g/kg diet (- PR vs. + PR), CS challenge of 18°C (- CS vs. + CS), and their interactions (PR × CS). Variables related to fish growth, redox status, and immune response were examined using the 2-way ANOVA in the general linear model (GLM) procedures. The means, pooled standard error of means (SEM), and *p*-values were shown for PR, CS, and PR×CS effects. The level of significance was set at *p*-value < 0.05. Whenever there was a statistically significant interaction effect, one-way ANOVA with Duncan’s multiple range test was run separately to rank the interaction means.

## Results

### Propolis analysis

Table 2 presents the results obtained from the PR analysis. It was found that the PR used in the present study contained high amounts of flavonoids (233.5 mg QE) and polyphenols (162.4 mg GAE) per g DM. Interestingly, the PR’s antioxidant activity was high, recording an IC50 of 79.3 μg/mL.

**Table 2:** The propolis chemical analysis.

Assay	Result ^1^
**DM (%)**	90.8 ± 5.07
**Carbohydrate (%)**	1.9 ± 0.07
**Crude fiber (%)**	68.7 ± 3.26
**Total lipids (%)**	9.2 ± 1.31
**Crude protein (%)**	2.6 ± 0.18
**Total ash (%)**	0.9 ± 0.04
**Phenolic content (mg GAE/g)** ^**2**^	162.4 ± 4.75
**Flavonoid content (mg QE/g)** ^**2**^	233.5 ± 9.82
**Antioxidant activity (IC50, μg/mL)** ^**2**^	79.3 ± 2.36

^1^ The mean ± standard deviation of three determinations on dry matter (DM) basis.

^2^ GAE, gallic acid equivalent; QE, quercetin equivalent; IC50, the sample concentration that achieved 50% inhibition of the 2,2-diphenyl-1-picrylhydrazyl (DPPH) free radicals.

### Preliminary experiment results

**Fig 2:**
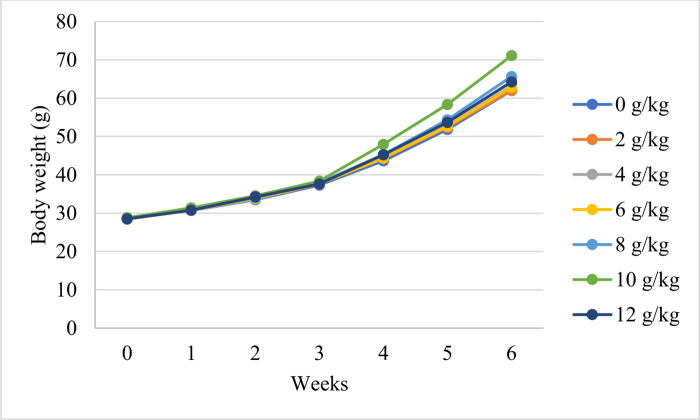
Weekly body weight of Nile tilapia fed on different levels of propolis for 6 consecutive weeks. The results indicate that body weights at 4, 5, and 6 weeks were significantly (*p* < 0.05) higher in the group fed on 10 g/kg propolis than in the other groups.

**Fig 3:**
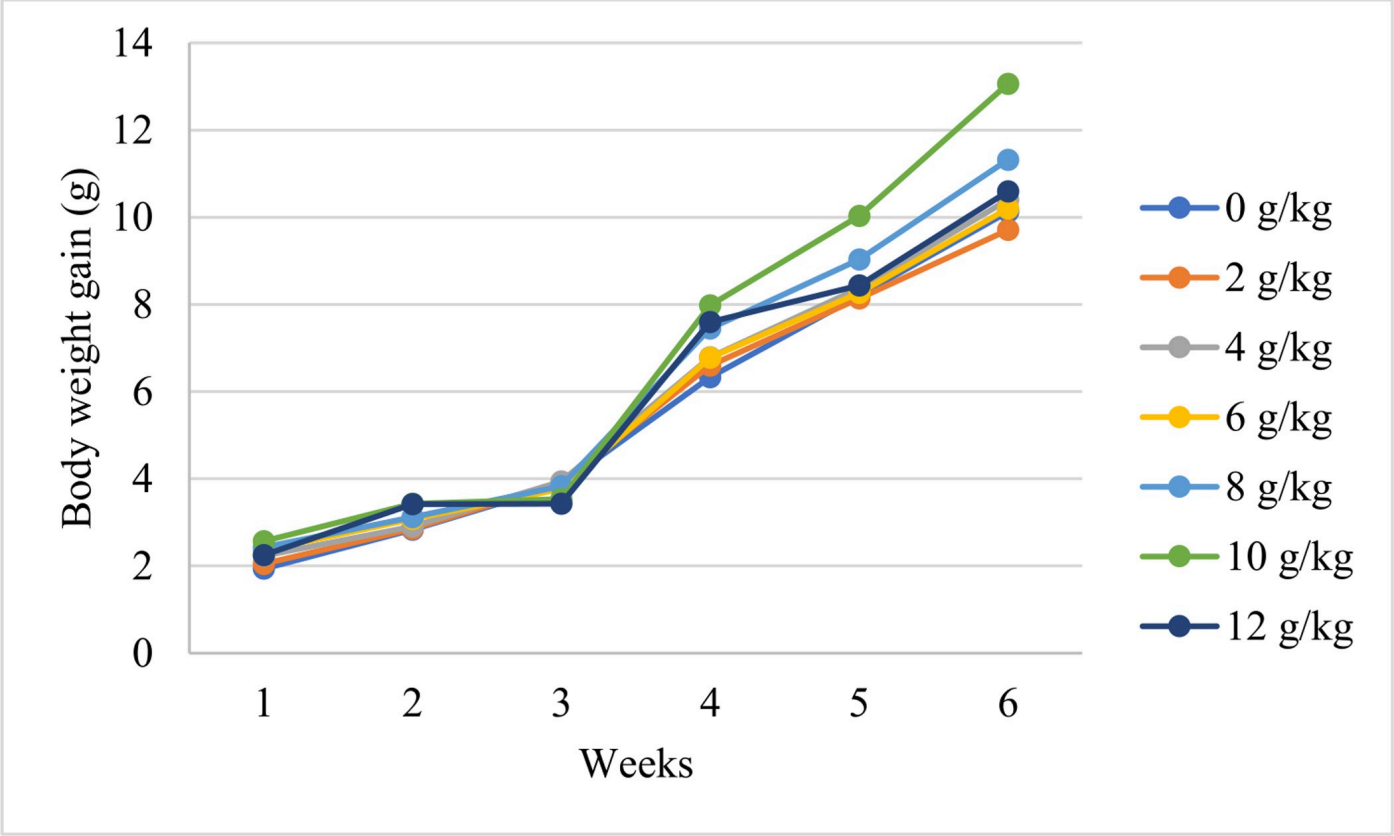
Weekly body weight gain of Nile tilapia fed on different levels of propolis for 6 consecutive weeks. The results indicate that body weight gains at 4, 5, and 6 weeks were significantly (*p* < 0.05) higher in the group fed on 10 g/kg propolis than in the other groups.

The results of preliminary study are shown in Figs 2 and 3. The results indicate that PR concentrations did not affect the fish’s body weights or body weight gains during the first three weeks of PR feeding (*p* > 0.05). From the 4^th^ to 6^th^ week of PR feeding, a linear effect (*p* < 0.05) for increasing the PR concentration was observed on the fish body weights and body weight gains. However, the fish’s body weights and body weight gains after PR feeding for 4, 5, and 6 weeks were significantly (*p* < 0.05) higher in the group fed on 10 g/kg propolis than in the other groups.

### Growth performance

The effect of dietary PR inclusion on the growth performance of Nile tilapia challenged by CS is shown in Table 3. Fish challenged by cold stress (+CS group) expressed a significant (*p* < 0.05) decrease in the FBW, BWG, SGR, and FI by approximately 19, 55, 50, and 12%, respectively, and a significant increase in the FCR by 2.1-fold and CMR by 29%, compared to non-challenged fish (-CS group). On the contrary, fish supplemented with propolis (+PR group) expressed a significant (*p* < 0.05) improvement in all growth performance parameters compared to the -PR fish group. Furthermore, there was a significant (*p* < 0.05) interaction effect for CS×PR on FI and CMR, while no interaction effect was found on the other growth traits. PR supplementation in the CS-challenged fish (+CS+PR group) significantly (*p* < 0.05) alleviated the FI reduction by 38% and the CMR elevation by 67%, compared to the CS-challenged group without PR (+CS-PR group).

**Table 3:** Effect of dietary propolis (PR) inclusion on the growth performance of Nile tilapia challenged by cold stress (CS) conditions.

Treatment groups	IBW (g)	FBW (g)	BWG (g)	SGR (%)	FI (g)	FCR	CMR (%)
**CS effect**							
**-CS**	29.85	46.24 ^a^	16.39 ^a^	1.56 ^a^	29.00 ^a^	1.83 ^b^	0.00 ^b^
**+CS**	30.00	37.41 ^b^	7.41 ^b^	0.78 ^b^	25.46 ^b^	3.85 ^a^	28.75 ^a^
**SEM**	0.156	0.372	0.395	0.036	0.290	0.289	1.029
***P*-value**	0.503	<0.001	<0.001	<0.001	<0.001	<0.001	<0.001
**PR effect**							
**-PR**	30.000	39.18 ^b^	9.18 ^b^	0.93 ^b^	24.48 ^b^	3.36 ^a^	21.67 ^a^
**+PR**	29.850	44.47 ^a^	14.62 ^a^	1.40 ^a^	29.98 ^a^	2.32 ^b^	7.08 ^b^
**SEM**	0.156	0.372	0.395	0.036	0.290	0.289	1.029
***P*-value**	0.503	<0.001	<0.001	<0.001	<0.001	0.019	<0.001
**CS×PR effect**							
**-CS-PR**	29.93	43.13	13.21	1.31	27.54 ^b^	2.09	0.00 ^c^
**-CS+PR**	29.78	49.35	19.58	1.81	30.46 ^a^	1.57	0.00 ^c^
**+CS-PR**	30.08	35.23	5.16	0.56	21.42 ^c^	4.63	43.33 ^a^
**+CS+PR**	29.93	39.59	9.67	1.00	29.50 ^a^	3.08	14.17 ^b^
**SEM**	0.220	0.526	0.559	0.051	0.410	0.408	1.455
***P*-value**	1.000	0.093	0.112	0.564	<0.001	0.219	<0.001

Data express the means, standard error of means (SEM), and probability (*P*-value) for each variable as affected by cold stress (CS), propolis (PR), and their interaction (CS×PR). Means with different superscripts within a treatment group in the same column significantly differ at *P* < 0.05. Abbreviations: -CS, fish group that was not challenged by cold stress; +CS, fish group that was challenged by cold stress; -PR, fish group that received a basal diet with no propolis supplementation; +PR, fish group that received a basal diet with 10 g/kg propolis; IBW, initial body weight; FBW, final body weight; BWG, body weight gain; SGR, specific growth rate; FI, feed intake; FCR, feed conversion ratio; CMR, cumulative mortality rate.

### Redox status

The effect of dietary PR inclusion on the redox status of Nile tilapia challenged by CS is presented in Table 4. The results demonstrated that the GSH, SOD, and CAT activities were significantly (*p* < 0.05) decreased by approximately 29, 41, and 38%, respectively, in the +CS challenged group compared to -CS non-challenged group. In contrast, the levels of MPO and MDA were significantly (*p* < 0.05) increased by 54 and 126%, respectively, in the +CS group compared to -CS group. Irrespective of CS challenge, it was found that PR treatment significantly (*p* < 0.05) improved the redox status of fish in the +PR group compared to the -PR group. Furthermore, PR treatment significantly (*p* < 0.05) alleviated the negative impact of CS on the GSH, SOD, CAT, and MDA by 30, 25, 19, and 37%, respectively, in the +CS+PR group compared to the +CS-PR group.

**Table 4:** Effect of dietary propolis (PR) inclusion on the redox status of Nile tilapia challenged by cold stress (CS) conditions.

Treatment groups	GSH (μmol/L)	SOD (U/mL)	CAT (U/mL)	MPO (U/L)	MDA (μmol/L)
**CS effect**					
**-CS**	16.35 ^a^	9.30 ^a^	79.68 ^a^	52.28 ^b^	6.91 ^b^
**+CS**	11.55 ^b^	5.52 ^b^	49.29 ^b^	80.34 ^a^	15.58 ^a^
**SEM**	0.151	0.102	0.584	0.547	0.372
***P*-value**	<0.001	<0.001	<0.001	<0.001	<0.001
**PR effect**					
**-PR**	11.99 ^b^	6.15 ^b^	54.42 ^b^	72.63 ^a^	13.96 ^a^
**+PR**	15.90 ^a^	8.67 ^a^	74.55 ^a^	59.99 ^b^	8.53 ^b^
**SEM**	0.151	0.102	0.584	0.547	0.372
***P*-value**	<0.001	<0.001	<0.001	<0.001	<0.001
**CS×PR effect**					
**-CS-PR**	13.92 ^b^	7.38 ^b^	63.89 ^b^	58.43	8.80 ^c^
**-CS+PR**	18.78 ^a^	11.22 ^a^	95.46 ^a^	46.14	5.03 ^d^
**+CS-PR**	10.06 ^d^	4.92 ^d^	44.94 ^d^	86.83	19.12 ^a^
**+CS+PR**	13.03 ^c^	6.13 ^c^	53.64 ^c^	73.85	12.04 ^b^
**SEM**	0.214	0.144	0.825	0.774	0.526
***P*-value**	<0.001	<0.001	<0.001	0.655	0.002

Data express the means, standard error of means (SEM), and probability (*P*-value) for each variable as affected by cold stress (CS), propolis (PR), and their interaction (CS×PR). Means with different superscripts within a treatment group in the same column significantly differ at *P* < 0.05. Abbreviations:—CS, fish group that was not challenged by cold stress; + CS, fish group that was challenged by cold stress;—PR, fish group that received a basal diet with no propolis supplementation; + PR, fish group that received a basal diet with 10 g/kg propolis; GSH, reduced glutathione; SOD, superoxide dismutase; CAT, catalase; MPO, myeloperoxidase; MDA, malondialdehyde.

### Immune response

The effect of dietary PR supplementations on the immune response of Nile tilapia challenged by CS is shown in Table 5. Compared with unchallenged fish (-CS group), CS challenge significantly (*p* < 0.05) impaired all the examined immunological measurements in the +CS group, indicating an increase of 20% in the H/L ratio and a decrease of 33, 42, 43, and 41% in the TWBC, PBL-SI, PA, and LA, respectively. On the contrary, PR supplementation significantly decreased the H/L ratio by 17% and increased the TWBC, PBL-SI, PA, and LA by 16, 67, 93, and 26%, respectively, in the +PR group compared to -PR group. Moreover, the CS×PR interaction had a significant (*p* < 0.05) effect on the H/L ratio, PBL-SI, and PA measurements. Compared to the +CS-PR group, the H/L ratio elevation was alleviated by 19% while the PBL-SI and PA reduction were modulated by 176% and 117% in the +CS+PR group.

**Table 5:** Effect of dietary propolis (PR) inclusion on the immune response of Nile tilapia challenged by cold stress (CS) conditions.

Treatment groups	TWBC (10^3^/μL)	H/L ratio	PBL-SI	PA (%)	LA (U/mL)
**CS effect**					
**-CS**	5.96 ^a^	0.44 ^b^	3.67 ^a^	20.78 ^a^	22.97 ^a^
**+CS**	3.96 ^b^	0.52 ^a^	2.12 ^b^	11.78 ^b^	13.54 ^b^
**SEM**	0.151	0.102	0.584	0.547	0.372
***P*-value**	<0.001	<0.001	<0.001	<0.001	<0.001
**PR effect**					
**-PR**	4.59 ^b^	0.52 ^a^	2.17 ^b^	11.12 ^b^	16.14 ^b^
**+PR**	5.33 ^a^	0.44 ^b^	3.62 ^a^	21.43 ^a^	20.37 ^a^
**SEM**	0.091	0.005	0.053	0.276	0.360
***P*-value**	<0.001	<0.001	<0.001	<0.001	<0.001
**CS×PR effect**					
**-CS-PR**	5.686	0.47 ^b^	3.20 ^b^	14.81 ^c^	20.57
**-CS+PR**	6.227	0.41 ^c^	4.14 ^a^	26.74 ^a^	25.37
**+CS-PR**	3.500	0.58 ^a^	1.13 ^c^	7.43 ^d^	11.71
**+CS+PR**	4.426	0.47 ^b^	3.11 ^b^	16.13 ^b^	15.37
**SEM**	0.129	0.007	0.075	0.391	0.509
***P*-value**	0.142	<0.001	<0.001	<0.001	0.266

Data express the means, standard error of means (SEM), and probability (*P*-value) for each variable as affected by cold stress (CS), propolis (PR), and their interaction (CS×PR). Means with different superscripts within a treatment group in the same column significantly differ at *P* < 0.05. Abbreviations:—CS, fish group that was not challenged by cold stress; + CS, fish group that was challenged by cold stress;—PR, fish group that received a basal diet with no propolis supplementation; + PR, fish group that received a basal diet with 10 g/kg propolis; TWBC, total white blood cells; H/L ratio, heterophil to lymphocyte cell ratio; PBL-SI, peripheral blood leukocyte stimulation index; PA, phagocytic activity; LA, lysozyme activity.

## Discussion

Although propolis supplementation’s impact on fish performance has been the subject of several research studies, information about how propolis affects tilapia’s ability to endure cold stress is insufficient. According to previous studies [6, 24], increasing propolis levels in fish diets up to 4 g/kg linearly improved fish growth, and positive effects on performance were exerted when 10 g/kg PR was used in fish nutrition. Therefore, a preliminary experiment was carried out before conducting the present study to determine the appropriate rate of PR within the range of 0–12 g/kg that induce notable effects on the growth performance of Nile tilapia. Based on the results of the preliminary experiment (Figs 2 and 3), the present work was designed to assess the potential effect of PR inclusion at 10 g/kg into fish diets for 4 weeks on the growth performance, redox status, and immune response of Nile tilapia challenged by CS conditions.

Exposure of fish to stress disturbs homeostasis and evokes some physiological reactions to maintain body balance and survivorship [9]. Indeed, Nile tilapia can survive and grow ideally in a wide range of water temperatures within 24–32°C, depending on the fish species, age, and genetic variations [43]. The current study manifested that CS-challenge at 18°C deteriorates all growth indices and feed efficiency of the Nile tilapia fish. The depression in feed intake reached 12% in the CS-challenged fish compared to the non-CS-challenged fish (Table 3). The decrease in feed intake may be the direct reason for the impairment in the other growth indices of tilapia, like FBW, BWG, SGR, and FCR [44]. Similar results were obtained in previous studies when Nile tilapia were reared at a chilly suboptimal temperature of 22°C rather than the ideal temperature of 28°C [45, 46]. In addition, CS induced a high mortality rate of approximately 43% during the experimental period in this study (Table 3). In previous studies on Nile tilapia, exposure to 18°C for 10–30 days negatively affected the fish survival and induced 20–60% mortality rates [12, 42].

Our results demonstrated that CS-induced high levels of MPO and MDA and low GSH, SOD, and CAT enzyme activity (Table 4). These results indicate that cold exposure caused high oxidative stress and low antioxidant defense in fish [47]. On the other hand, our results indicated a protective interaction effect for propolis against the negative impacts of CS on redox status of Nile tilapia fish. The PR supplementation in the +CS+PR fish group reduced 37% in the plasma MDA compared to the +CS-PR fish group. These results agree with previous reports confirming that PR alleviates the thermal stress experience in Nile tilapia fish [6]. Propolis was found to support the antioxidant enzyme system, as the PR treatment alleviated the reduction in GSH, SOD, and CAT by CS challenge. The ameliorative effect of PR via an antioxidant pathway was also reported in Nile tilapia exposed to oxidative stress induced by cyanobacterium toxins [25]. These effects could be attributed to the high flavonoids and polyphenolic contents and PR’s antioxidant activity, as shown in Table 2. Similarly, reducing the oxidative damage and improving the antioxidant capacity was induced by polyphenols extracted from green tea in grass carp [48] or from *Annona squamosa* leaf in Nile tilapia [42]. It was suggested that the presence of phytochemical components, including flavonoids, polyphenols, saponins, tannins, sesquiterpenes, anthocyanins, terpenoids, and quinones, in propolis enhances the antioxidant potency and blocks the free radical production in biological systems [49–51].

Furthermore, the immunological parameters were dramatically reduced in fish challenged by CS, except for the H/L ratio (Table 5). The increase in the H/L ratio could be considered an indicator of stress in fish exposed to CS [52]. The negative impact of CS on fish immunity has been previously explored on the T and B lymphocytes, antibodies, and humoral response [53]. The present study indicated a significant interaction between PR and CS for the immune response of Nile tilapia. Propolis supplementation to CS-challenged fish reduced 19% in the H/L ratio and enhanced more than 100% of the other immunological parameters such as PBL-SI and PA (Table 5). In line with previous studies, propolis was reported to modulate the immunity of poultry [21, 54–57] and fish [6, 24, 28]. Propolis’ antioxidant qualities have been hypothesized to boost leukocyte cell viability via modulating a cascade of fork-head box (Foxo) genes involved in the resistance to apoptosis and oxidative damage [58, 59]. In addition, it was reported that PR extracts enhanced the lymphocyte proliferation and monocyte-macrophage function (phagocytic activity) during bacterial infection in Nile tilapia [24] and gilthead seabream [60]. Such immunological effects for PR could be attributed to some compounds identified in propolis, such as pinocembrin, galanin, pinobanksin, pinobanksin-3-acetate, and caffeic acid esters [61]; however, these compounds were not analyzed in the present study.

Consequently, the positive impacts of propolis on the antioxidant enzymes and immunological parameters in the CS-challenged Nile tilapia are also reflected in their growth indices. The FI was 38% higher in the +CS+PR group than in the +CS-PR group. Also, the fish mortality reduced from 43% to 14% in the same groups, respectively. The improvement of these two parameters by PR may be due to propolis’s antimicrobial activity, which, in turn, improves intestinal digestion and absorption [62]. Thus, PR increased the FBW, BWG, and SGR by 12%, 88%, and 78%, respectively, in fish challenged with CS (Table 3). It was also suggested that PR contributed to the increase of growth performance in CS-challenged fish through supporting metabolism and digestion-coenzyme factors [15, 63].

## Conclusions

The dietary propolis supplementation at 10 g/kg level improved the growth performance, redox status, and immune response of Nile tilapia fish reared at 26°C. In contrast, exposure of Nile tilapia to cold stress impairs their growth performance, redox status, and immune response. Propolis supplementation to fish challenged with cold stress can alleviate the negative effects of cold stress on fish weights, specific growth rates, feed efficiency, antioxidant enzyme activity, lymphocyte proliferation, and phagocytosis activity. Moreover, propolis treatment can reduce stress indicators such as H/L ratio, malondialdehyde, and mortality rates in fish exposed to cold stress. Therefore, adding propolis at a rate of 10 g/kg to the diet of Nile tilapia fish could be recommended as a potential nutritional strategy to improve their performance, especially under cold-stress conditions.

## Supporting information

S1 Data set (RAR)
